# Three Immune-Associated Subtypes of Diffuse Glioma Differ in Immune Infiltration, Immune Checkpoint Molecules, and Prognosis

**DOI:** 10.3389/fonc.2020.586019

**Published:** 2020-12-23

**Authors:** Quanwei Zhou, Xuejun Yan, Weidong Liu, Wen Yin, Hongjuan Xu, Damei Cheng, Xingjun Jiang, Caiping Ren

**Affiliations:** ^1^ Department of Neurosurgery, Xiangya Hospital, Central South University, Changsha, China; ^2^ Cancer Research Institute, School of Basic Medical Science, Central South University, Changsha, China; ^3^ The NHC Key Laboratory of Carcinogenesis and The Key Laboratory of Carcinogenesis and Cancer Invasion of the Chinese Ministry of Education, Xiangya Hospital, Central South University, Changsha, China

**Keywords:** diffuse glioma, immune checkpoint molecule, tumor immune infiltration, bioinformatic analysis, The Cancer Genome Atlas (TCGA), Gene Expression Omnibus (GEO), immune-associated subtype

## Abstract

Diffuse glioma is one of the most prevalent malignancies of the brain, with high heterogeneity of tumor-infiltrating immune cells. However, immune-associated subtypes of diffuse glioma have not been determined, nor has the effect of different immune-associated subtypes on disease prognosis and immune infiltration of diffuse glioma patients. We retrieved the expression profiles of immune-related genes from The Cancer Genome Atlas (TCGA) (n = 672) and GSE16011 (n = 268) cohorts and used them to identify subtypes of diffuse glioma via Consensus Cluster Plus analysis. We used the limma, clusterProfiler, ESTIMATE, and survival packages of R for differential analysis, functional enrichment, immune and stromal score evaluation respectively in three subtypes, and performed log-rank tests in immune subtypes of diffuse glioma. The immune-associated features of diffuse glioma in the two cohorts were characterized via bioinformatic analyses of the mRNA expression data of immune-related genes. Three subtypes (C1–3) of diffuse glioma were identified from TCGA data, and were verified using the GSE16011 cohort. We then evaluated their immune characteristics and clinical features. Our mRNA profiling analyses indicated that the different subtypes of diffuse glioma presented differential expression profile of specific genes and signal pathways in the TCGA cohort. Patients with subtype C1, who were mostly diagnosed with grade IV glioma, had poorer outcomes than patients with subtype C2 or C3. Subtype C1 was characterized by a higher degree of immune cell infiltration as estimated by GSVA, and more frequent wildtype IDH1. By contrast, subtype C3 included more grade II and *IDH1*-mutated glioma, and was associated with more infiltration of CD4^+^T cells. Most subtype C2 had the features between subtypes C1 and C3. Meanwhile, immune checkpoints and their ligand molecules, including PD1/(PD-L1/PDL2), CTLA4/(CD80/CD86), and B7H3/TLT2, were significantly upregulated in subtype C1 and downregulated in subtype C3. In addition, patients with subtype C1 exhibited more frequent gene mutations. Univariate and multivariate Cox regression analyses revealed that diffuse glioma subtype was an effective, independent, and better prognostic factor. Therefore, we established a novel immune-related classification of diffuse glioma, which provides potential immunotherapy targets for diffuse glioma.

## Introduction

Diffuse glioma is by far the most prevalent type of primary central nervous system (CNS) malignant tumor, which accounts for >60% of all primary brain tumors (1). The 2016 World Health Organization (WHO) Classification of CNS tumors categorizes diffuse gliomas into WHO grades II, III, and IV glioma, including oligodendrogliomas, astrocytomas, oligoastrocytomas, and glioblastomas multiforme (GBMs). Almost all glioma patients, particularly those with GBM, exhibit recurrence despite being treated with the standard treatment consisting of maximal-safety surgical resection coupled with radiotherapy and chemotherapy (2, 3). The prognosis of patients with diffuse glioma varies significantly depending on the tumor grade, with median survival ranging from 1 to 15 years (4). GBM is one of the most aggressive tumors, with a median survival of approximately 14 months (5). By contrast, patients with grade II or III glioma have a relatively better prognosis, with a median survival of >7 years (6); however, with frequent disease progression, grade II–III gliomas recur as tumors with higher grades and become resistant to therapy (7). So far, traditional treatments alone have not improved patient prognosis, and novel treatment strategies for patients with diffuse glioma are warranted.

Studies have shown that the complexity of the tumor microenvironment can be attributed to diverse immune cells. Glioma cells have been shown to recruit a variety of tumor-infiltrating immune cells, including macrophages, myeloid suppressor cells, dendritic cells, neutrophils, T and B lymphocytes, and natural killer (NK) cells, into the tumor microenvironment (8, 9). In addition, the tumor microenvironment plays a crucial role in glioma progression (10), and is associated with clinical outcomes (11). As a critical component of the complex microenvironment, tumor-infiltrating immune cells play a vital role in inhibiting or promoting tumor progression (12). Both inter- and intratumoral heterogeneity of the immediate immune microenvironments are associated with differences in the number and type of immune cells, thus representing a significant obstacle to the treatment of diffuse glioma.

The distinct immune environment of the CNS should be considered in glioma patients scheduled for immunotherapy (13) because the blood–brain barrier shields the tumor cells from conventional treatments. Nonetheless, glioma proliferation can damage the blood–brain barrier, allowing the entry of tumor-related immune cells into the tumor tissue and establishment of an immunosuppressive state in glioma. The unique CNS environment may lead to development of resistance against immunotherapy in glioma cells. To address this problem, immune checkpoint inhibitors have been developed and these checkpoint inhibitors have shown superior clinical efficacy against various tumor types. Programmed death-1 (PD-1), an immune checkpoint protein, is significantly upregulated in tumor-infiltrating immune cells (14). The PD-1 receptor expressed on the surface of CD8^+^ T-cells binds to PD-L1 on the tumor cell surface, thereby inactivating the host T-cell immune responses. Theoretically, blocking the binding of PD-L1 to PD-1 should reverse their immune suppressive effect in tumors. Pembrolizumab, a humanized monoclonal antibody against PD-1, inhibits T-cell inactivation rather than recruiting T cells to infiltrate tumor (15). One study indicates that pembrolizumab is more effective in treating tumors with a large number of infiltrating T cells as compared to tumors with lower abundance of T cells (15). Furthermore, evidence has shown that the efficacy of immunotherapy varies considerably among individuals because of inter- and intratumoral heterogeneity of the immune microenvironment. Therefore, novel classification and treatment modalities based on immune profiles are required for patients with diffuse gliomas. In the present study, we utilized The Cancer Genome Atlas (TCGA) database to identify three consensus immune-associated subtypes (C1–3) of diffuse glioma by clustering analyses based on the expression of 2,498 immune-associated genes. We then analyzed their immune characteristics and clinical features and revealed significant differences in gene signatures, signaling pathways, immune infiltration, expression of immune checkpoint genes, and clinical outcomes of these cases. Moreover, univariate and multivariate Cox regression analyses indicated that the identified subtype classification is an effective independent prognostic factor.

## Materials and Methods

### Data Sources and Data Processing

High-throughput RNA sequencing (RNA-seq) data (n = 703), information on somatic mutations (MAF files) (n = 1,090), and clinical data (n = 1,105) from patients with diffuse gliomas were downloaded from TCGA (https://tcga-data.nci.nih.gov/tcga/). Two RNA-seq datasets, TCGA-lower grade glioma (LGG) and TCGA-GBM, were merged into a single metadataset, and batch effects were removed by applying the ComBat function in the SVA package of the R software (16). In the TCGA cohort, 677 samples, including five nontumor samples, 249 grade II samples, 262 grade III samples, and 161 grade IV samples, were selected from 703 cases by averaging the values from the same patient and excluding some samples. The GSE16011 dataset, involving 268 samples, was downloaded from database Gene Expression Omnibus (https://www.ncbi.nlm.nih.gov/gds/) (17). In addition, tumor mutational burden (TMB) and mutation counts were computed from somatic mutation frequencies. Sixteen samples with outlying values were excluded by means of the “outliers” package of R.

### Identification of Subtypes Based on Immune-Related Genes in Diffuse Glioma

We retrieved a list of 2,498 immune-related genes from ImmPort (https://www.immport.org/), a publicly available immunology data resource. Expression profiles of the immune-related genes were obtained by mining of TCGA and GSE16011 data and were used to identify diffuse glioma subtypes via Consensus Cluster Plus analysis (17). Before identifying the subtypes, we excluded genes not expressed in >80% of the samples, resulting in 1,106 candidate genes for cluster analysis. Consensus clustering was performed using the K-means algorithm with 1,000 resampling iterations by random selection of a group of the samples and the most variant probe sets (80%). The optimal cluster number was determined using the consensus matrix (CM) and CDF curves of the consensus score. T-distributed stochastic neighbor embedding (t-SNE) decreased the dimensionality of the features and was employed to validate the subtype assignment by means of mRNA expression data corresponding to immune-related genes (18).

### Differential Analysis and Comparative Functional Enrichment Analysis of the Subtypes

For each subtype, subtype-specific genes were identified by comparing the samples in a given subtype with the remaining samples using the “limma” package of R (19, 20). The cutoff criteria for differentially expressed genes were defined as false discovery rate (FDR) <0.05 and absolute log_2_FC (fold change) >1. Kyoto Encyclopedia of Genes and Genomes (KEGG) analysis of differentially expressed genes among diffuse glioma subtypes was performed using the clusterProfiler package (21). We selected the top 50 significant immune-related genes with the largest log_2_FC in each subtype as molecular markers of immune-associated subtypes.

### Estimation of Immune-Cell Abundance

We first compared immune and stromal scores evaluated in the three subtypes by means of the ESTIMATE package of R, which can calculate the abundance of stromal and immune cells (22). Relative abundance of 28 immune cell types in the tumor microenvironment was evaluated by gene set variation analysis (GSVA), which can estimate the scores of signaling pathways or signatures based on transcriptomic data (23). We retrieved the gene list corresponding to each immune cell type from a recent publication (24). Next, differential analysis of the relative abundance of 28 immune cell types was performed using the limma package of R, and the immune cells with absolute log_2_FC >1 (FDR < 0.05) were defined as subtype-specific immune cells. Furthermore, we determined differences in the abundance of activated CD8^+^ T cells, activated dendritic cells, activated CD4+ T cells, neutrophils, NK cells, and macrophages in the validation cohort. After that, we calculated the abundance of immune cells using the CIBERSORT algorithm from the Tumor IMmune Estimation Resource (TIMER) online database (cistrome.shinyapps.io/timer) (25, 26).

### Survival Analysis

We excluded the samples with <30 days of overall-survival data and with missing values of clinical features in the TCGA and GSE16011 datasets. Kaplan–Meier survival curves were plotted to estimate survival distributions for each subtype in the TCGA and GSE16011 datasets. We performed the log-rank test to assess the significance of differences between the identified subtypes using the survival package in R (27). Univariate Cox and backward stepwise multivariate Cox regression analyses were performed to identify independent prognostic factors among the following variables: age, gender, tumor grade, *IDH* status, and chromosome 1p/19q codeletion status. *P* <0.05 indicated statistical significance.

### Statistical Analysis

Unpaired Student’s *t* test was performed to compare two groups with normally distributed variables, while for comparisons of multiple groups, one-way analysis and Kruskal–Wallis tests of variance were conducted as parametric and nonparametric methods, respectively. Contingency variables were analyzed using the χ^2^ or Fisher’s exact tests. Pearson’s correlation coefficient was determined to assess the correlation between TMB and the mRNA expression of *PD-1* and *PD-L1* as well as between TMB and the abundance of immune cells. All statistical analyses were performed in GraphPad Prism v.7.0 or R (v.3.6.0, https://www.r-project.org/). A two-tailed *P* value <0.05 was considered significant.

## Results

### Identification of Three Subtypes in Diffuse Glioma

**Figure 1:**
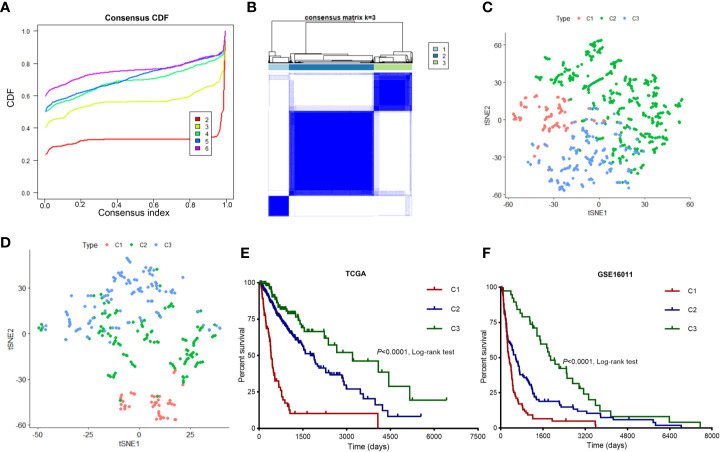
Identification of immune-related subtypes of diffuse glioma in TCGA cohort. **(A)** The cumulative distribution function (CDF) curves in consensus cluster analysis. Consensus scores for different subtype numbers (k = 2 to 6) are presented. **(B)** The heatmap illustrating the consensus matrix at k = 3 in TCGA cohort. **(C, D)** The stratification into three subtypes validated by t-SNE in TCGA and GSE16011 cohorts. Each dot represents a single sample, and each color denotes a subtype. **(E, F)** Survival analysis of patients with the three diffuse glioma subtypes (C1, C2, and C3) in TCGA and GSE16011 cohorts. The log-rank test was conducted to determine the significance of the differences.

We mined 1,106 candidate immune-related genes from TCGA-LGG and TCGA-GBM cohorts and clustered them into three diffuse glioma subtypes. We utilized Consensus Cluster Plus to identify the different subtypes (K = 2, 3, 4, 5, and 6) among 672 diffuse glioma samples. The optimal division (k = 3) was selected as the optimal number of clusters based on the CM and CDF curves of the consensus score (**Figures 1A, B**). At K = 3, the boundary of the CM heatmap remained relatively clear-cut, implying that the sample cluster was stable and robust. To validate the assignment of the subtypes, t-SNE was performed to decrease dimensionality. It was confirmed that two-dimensional t-SNE distribution patterns were robustly consistent with the subtype clustering (**Figure 1C**). This result implied that the three clusters of samples were successfully separated from each other. We then verified these three subtypes identified in TCGA cohort using the GSE16011 dataset as described above (**Figure 1D**). Using the previously mentioned K = 3 classification, we uncovered significant prognostic differences in TCGA cohort (log-rank test *P* < 0.0001, **Figure 1E**), with shorter median survival for subtype C1 (n = 92, median survival: 406 days) than for subtypes C2 (n = 374, median survival: 1,762 days, *P* < 0.001) and C3 (n = 163, median survival: 3,200 days, *P* < 0.001). Patients with diffuse glioma subtype C2 showed shorter median survival than patients with subtype C3 (log-rank test, P < 0.01; **Figure 1E**). These findings are consistent with the differences in survival among the three subtypes in the GSE16011 dataset (**Figure 1F**). Overall, based on the gene expression profiles, we identified three immune subtypes that were associated with clinical outcomes.

### Subtype-Specific Genes and Signaling Pathways in Diffuse Glioma

**Figure 2:**
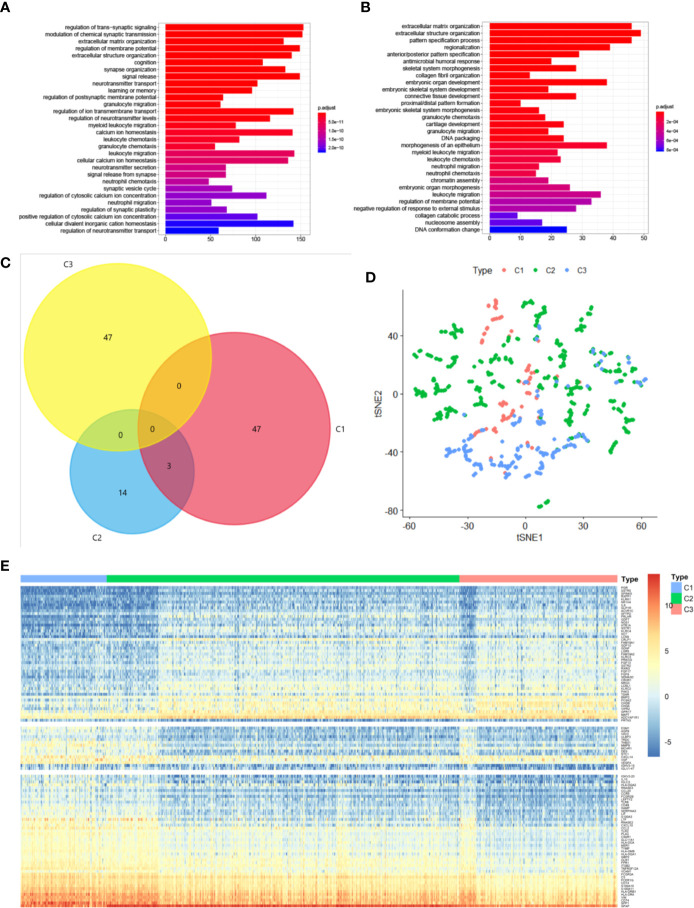
Subtype-specific genes and signaling pathways in diffuse glioma. **(A, B)** KEGG analysis of differentially expressed genes in subtypes C1 **(A)** and C3 **(B)**. The size and color of the dots represent the number of genes and the range of P values, respectively. **(C)** A Venn diagram depicting overlapping immune-gene sets among the three subtypes. **(D)** t-SNE analysis showing the stratification based on the expression profile of subtype-specific genes in TCGA cohort. **(E)** The expression profile of 109 subtype-specific genes.

We performed differential analyses to identify differentially expressed genes and signaling pathways specific to the three identified subtypes. We assumed genes to be significantly differentially expressed when absolute log_2_FC was >1.0 and FDR was <0.05 compared with the other subtypes. We then screened 4,293 genes, including 3,491 genes for subtype C1, 156 genes for subtype C2, and 646 genes for subtype C3; these were then selected for KEGG analyses to identify the specific signaling pathways. It was confirmed that these differentially expressed genes in subtypes C1 and C3 were enriched for terms associated with different biological processes; however, both were found to be enriched for terms associated with the neutrophil-related pathway, including neutrophil chemotaxis and migration (**Figures 2A, B**). The set of differentially expressed genes in subtype C2 was not enriched for terms associated with any pathway. Subtype-specific genes were considered differentially expressed in one subtype but not in other subtypes. The top 50 differentially expressed genes of each subtype were selected as candidate molecular markers. We identified 108 subtype-specific markers, with 47 specific genes for subtype C1, 14 for subtype C2, and 47 for subtype C3 (**Figure 2C**). We additionally conducted t-SNE to decrease the dimensionality of the features in TCGA cohort, in which the three sample clusters were separated from each other (**Figure 2D**). The 108 subtype-specific signature genes were found and are shown in **Figure 2E**. The genes were differentially expressed in the three subtypes, indicating that these genes are significant indicators of these subtypes. Overall, we successfully identified signature genes and signature signaling pathways of the three subtypes.

### Characteristics of the Three Subtypes of Diffuse Glioma

**Figure 3:**
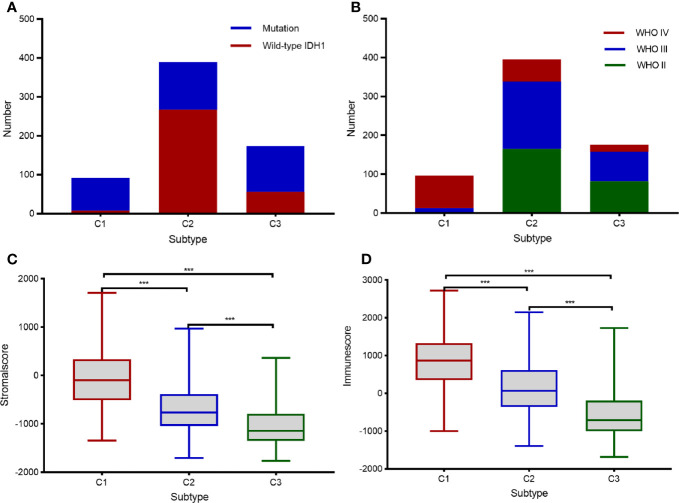
Clinical characteristics of the three subtypes in TCGA cohort. **(A, B)** Histogram depicting the frequency of *IDH* mutations in the tumor **(A)** and various tumor grades **(B)** in each subtype. **(C, D)** Box plots displaying the immune scores **(C)** and stromal scores **(D)** in each subtype. Pairwise comparisons of the subtypes were performed using Student’s *t*-test, and *P* <0.05 was regarded as significant. *** means P<0.001.

To identify clinical characteristics of the three subtypes of diffuse glioma, we examined conventional clinical variables, including age, gender, tumor grade, *IDH* mutation status, 1p19q codeletion status, and *MGMT* promoter status in TCGA cohort. The results indicated that the clinical characteristics differed significantly among the three immune subtypes of diffuse glioma (**Table 1**; χ^2^ test, *P* < 0.05). In this study, subtype C1 had a higher histological grade than the other subtypes (χ^2^ test, FDR < 0.0001). Mutation status of *IDH*, including *IDH1*, is a distinctive biomarker for glioma classification and prognostic assessment (28). The proportion of cases with wild-type *IDH* in the tumor was significantly higher in subtype C1 than in the other subtypes (χ^2^ test, FDR < 0.0001, **Figure 3A**). These findings further explained why patients with subtype C1 had a dismal prognosis relative to the patients with other subtypes. In contrast, subtype C3 contained a slightly higher proportion of cases of WHO grade II than did the other subtypes (χ^2^ test, FDR < 0.0001); meanwhile, the proportion of cases with mutant *IDH1* in the tumor was significantly higher in subtype C3 than in subtypes C1 and C2 (χ^2^ test, FDR < 0.0001, **Figures 3A, B**). The distribution of tumor grades and *IDH1* mutation states in subtype C2 was intermediate between subtypes C1 and C3. At the same time, samples with the same grade were distributed across different subtypes, indicating heterogeneity of the same grade of glioma. Since the classification was based on immune-related genes, we explored the characteristics of the microenvironment. The ESTIMATE approach was employed to infer the stromal- and immune-cell admixture using TCGA transcriptome data on diffuse glioma (22, 29). The stromal scores and immune scores were significantly higher (*P* < 0.001) in subtype C1 than those in the other subtypes (**Figures 3C, D**). Conversely, the stromal and immune scores were significantly lower in subtype C3 than in the other subtypes. Similarly, the characteristics of subtype C2 diffuse glioma were intermediate between subtypes C1 and C3 (**Figures 3C, D**). Overall, the characteristics of the three subtypes of diffuse glioma varied significantly.

**Table 1:** Association of the three immune-related with clinical characteristics in TCGA cohort.

Clinical Characteristics		Molecular subtypes (n, %)			*p* value
		C1(97,14.4)	C2(397,59.1)	C3(178,26.5)	
Age					< 0.0001
	<40y	14(14.4)	139(35.0)	71(40.0)	
	>=40y	82(84.5)	215(54.2)	92(51.7)	
	N/A	1(1.0)	43(10.8)	15(8.4)	
Gender					0.012
	Female	32(33.0)	151(38.0)	71(40.0)	
	Male	64(66.0)	203(51.1)	91(51.1)	
	N/A	1(1.0)	43(10.8)	15(8.4)	
Histology					< 0.0001
	Astrocytoma	5(5.2)	128(32.2)	34(19.1)	
	Oligodendroglioma	5(5.2)	87(21.9)	82(46.1)	
	Oligoastrocytoma	2(2.1)	81(20.4)	29(16.3)	
	Glioblastoma	84(86.6)	58(14.6)	18(10.1)	
	N/A	1(1.0)	43(10.8)	15(8.4)	
Grade					< 0.0001
	G2	1(1.0)	143(36.0)	72(40.4)	
	G3	11(11.3)	153(38.5)	73(41.0)	
	G4	84(86.6)	58(14.6)	18(10.1)	
	N/A	1(1.0)	43(10.8)	15(8.4)	
IDH status					< 0.0001
	Mutant	80(82.5)	115(29.0)	43(24.2)	
	WT	11(11.3)	279(70.3)	135(75.8)	
	N/A	6(6.2)	3(0.8)	0(0)	
1p/19q codeletion					< 0.0001
	Non-codel	2(2.1)	89(22.4)	77(43.3)	
	Codel	93(95.8)	304(76.6)	101(56.7)	
	N/A	2(2.1)	4(1.0)	0(0)	
MGMT promoter status					< 0.0001
	Unmethylated	44(45.3)	93(23.4)	27(15.2)	
	Methylated	32(33.0)	296(74.6)	146(82.0)	
	N/A	21(21.6)	8(2.0)	5(2.8)	

y, years; WT, wild-type; N/A, not available.

### Correlation of Diffuse Glioma Subtypes With Immune Infiltration in The Cancer Genome Atlas Cohort

**Figure 4:**
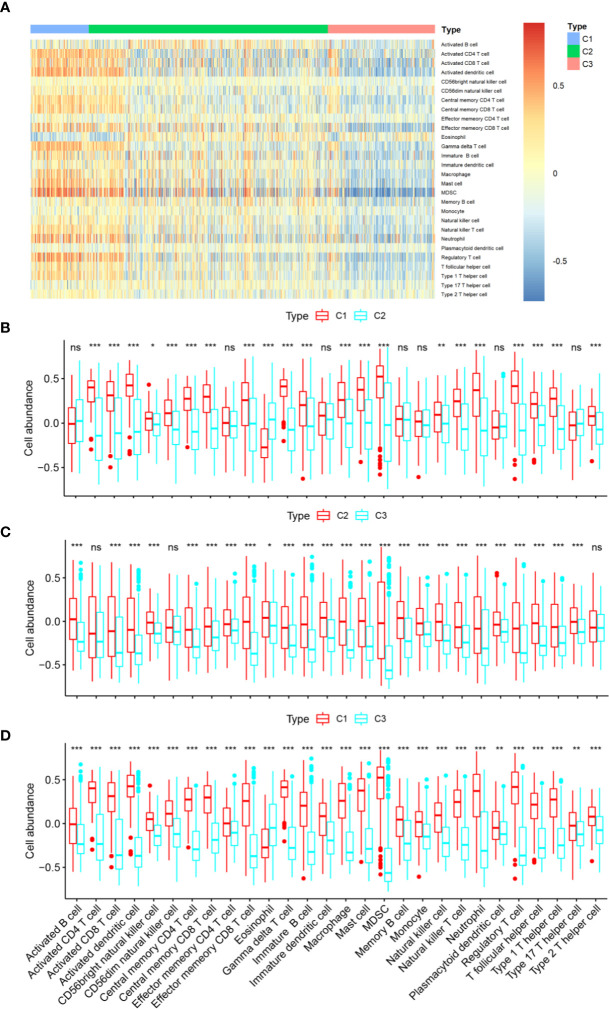
Immune characteristics of the three subtypes in TCGA cohort. **(A)** The heatmap showing the abundance of immune-cell populations calculated by GSVA in the three subtypes. **(B**–**D)** A box plot depicting differences in immune infiltration in C1 **(B)**, C2 **(C)**, and C3 **(D)**. Unpaired Student’s *t*-test was performed to compare two groups with normally distributed variables, and the P values are labeled above each box plot with asterisks (“ns” means “not significant,” *P < 0.05, **P < 0.01, ***P < 0.001).

**Figure 5:**
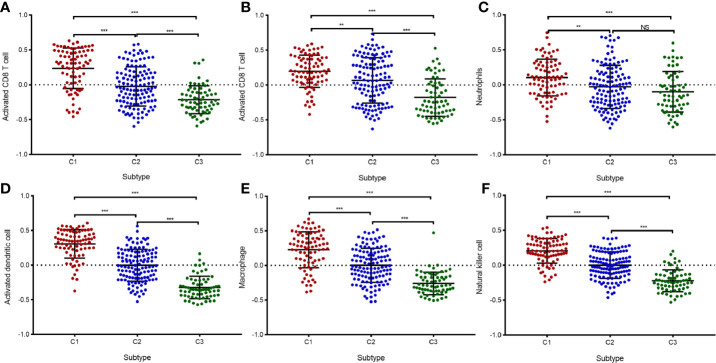
Validation of immune infiltration data from the three immune-related in the GSE16011 cohort. **(A**–**F)** A box plot displaying differential abundance of CD8^+^ T cells **(A)**, activated CD4^+^ T cells **(B)**, neutrophils **(C)**, activated dendritic cells **(D)**, macrophages **(E)**, and NK cells **(F)** among subtypes C1, C2, and C3. The pairwise comparisons between subtypes were performed using the Student’s *t*-test, and *P* <0.05 was considered significant. **, *** means P<0.01 and P<0.001, respectively. “ns” means “not significant”.

Given the significant differences in immune scores among the three identified subtypes of diffuse glioma, we then investigated whether such subtypes are associated with immune infiltration. First, relative presence of 28 immune cell types was quantified using the GSVA package in R as previously described (24), and the results were presented in a heatmap (**Figure 4A**). After that, differential analyses were conducted to identify subtype-specific immune cells, which were defined as immune cells with a higher GSVA score in a given subtype relative to the other subtypes. There were significant differences in most of immune cell types between subtype C1 and the other subtypes (**Figures 4B, D**). Moreover, there were significantly higher abundance of 27 immune cell populations, except for eosinophils, in C1 compared with C2 or C3 (**Figures 4B, D**). In contrast, subtype C3 exhibited a reduction in these 27 immune cell populations and greater enrichment of eosinophils (**Figures 4C, D**). We also noted higher abundance of 25 immune cell types in subtype C2 relative to subtype C3 (**Figure 4B**). We next assessed the abundance of six cell types—activated CD4^+^ T cells, activated CD8^+^ T cells, NK cells, macrophages, activated dendritic cells, and neutrophils—in the GSE16011 cohort (**Figure 5**). Consequently, the six immune cell populations were significantly more abundant in subtype C1 relative to subtypes C2 and C3. On the other hand, the abundance of these six immune cell populations was remarkably less in subtype C3 compared with that in the other subtypes. Macrophages play an important role in glioma maintenance and progression. By contrast, macrophages of the M1 phenotype (they have a potential to suppress tumor growth) are usually not present at a tumor site (30). We then investigated the correlation of the diffuse glioma subtypes with macrophage subtypes in TCGA cohort using the CIBERSORT algorithm from the TIMER online database. We found significant differences between C1 and the other subtypes, with the highest abundance of M2 macrophages in C1 and the lowest in C3 (**Supplementary Figure 2**). In addition, the CIBERSORT algorithm did not identify a significant difference in M1 enrichment among the three subtypes (**Supplementary Figure 2**). These results are in agreement with the immune- and stromal-score findings. Therefore, there were differences in the abundance of immune cells among the three subtypes according to the differences in immune-related genes.

### Differential Expression of Immune Checkpoint Molecules and Tumor Mutational Burden in The Cancer Genome Atlas Cohort

**Figure 6:**
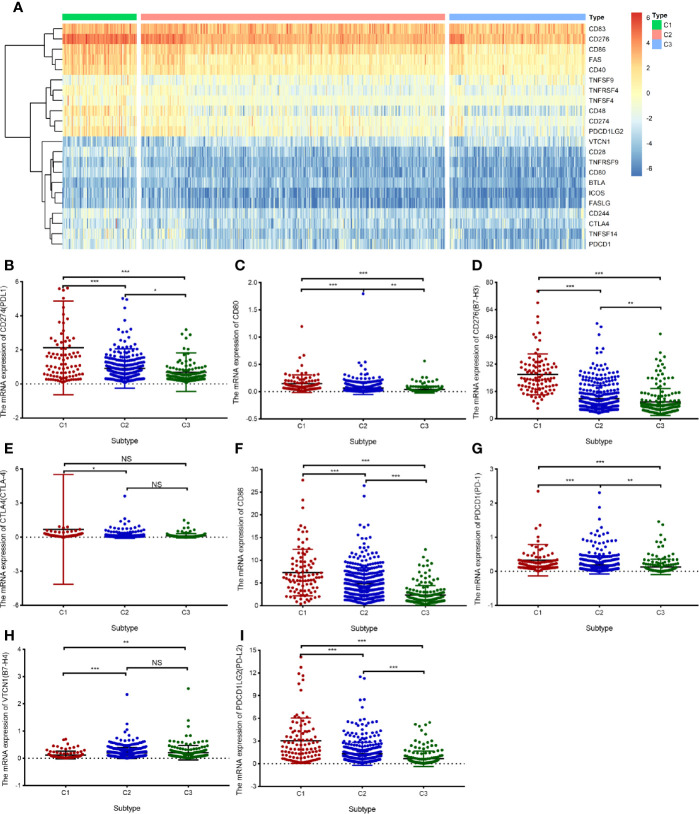
Differential expression of immune checkpoint genes in the TCGA cohort. **(A)** The heatmap illustrating mRNA expression of 16 immune checkpoint genes in three subtypes. **(B**–**I)** A box plot displaying the differential abundance of PD-L1 **(B)**, CD80 **(C)**, B7-H3 **(D)**, CTLA-4 **(E)**, CD86 **(F)**, PD-1 **(G)**, B7-H4 **(H)**, and PD-L2 **(I)** among subtypes C1, C2, and C3. The pairwise comparisons between subtypes were performed using Student’s *t*-test, and *P* <0.05 was considered significant. “ns” means “not significant”, *P < 0.05, **P< 0.01, ***P < 0.001.

**Figure 7:**
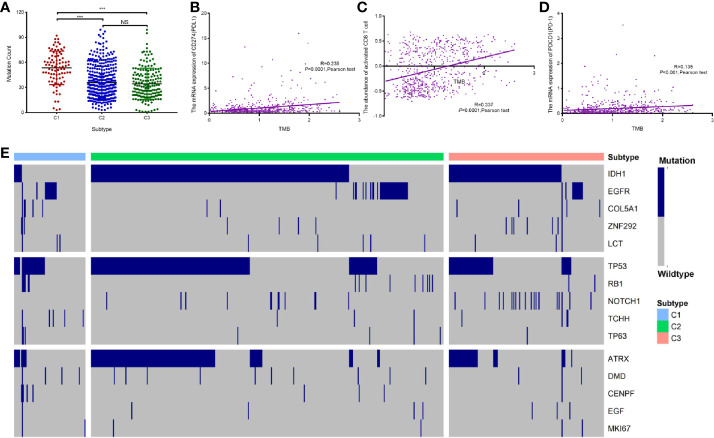
Association between immune-related subtypes and somatic mutations in TCGA cohort. **(A)** The number of mutations in diffuse glioma subtypes. **(B**–**D)** A scatter plot depicting the correlation between TMB and PD-L1 mRNA expression **(B)**, relative abundance of CD8^+^ T cells **(C)**, and PD-1 mRNA expression **(D)**. **(E)** Oncoprint analysis of mutation status of genes involved in “glycolysis,” “P53 pathway,” and “G2/M checkpoint”. “ns” means “not significant”, *** means P<0.001.

In the three subtypes, we investigated the expression of immune checkpoint genes that perform crucial functions in cellular immune regulation (31). Similar to the abundance of immune cells, the expression of most checkpoint genes, such as *PD1*, *PD-L1*, *CTLA4*, and *B7H3*, was found to be upregulated in subtype C1 compared with that in the other subtypes. By contrast, the expression of most checkpoint genes was downregulated in subtype C3 compared with that in the other subtypes. In addition, the expression of ligands corresponding to the checkpoint molecules, such as PD-L1/PD-L2 for PD1, CD86/CD80 for CTLA4, and TLT2 for B7H3, was significantly upregulated in subtype C1 compared with that in the other subtypes (**Figure 6**). The expression of *B7H4*, one of the checkpoint genes, was markedly downregulated in subtype C1 compared with that in subtypes C2 and C3 (**Figure 6H**). The expression of PD-L1 and numbers of tumor-infiltrating immune cells have been reported to be increased in GBM (32), in line with our findings. Another study has shown that TMB correlates with antitumor immunity and responses to immune checkpoint blockade in multiple tumor types (33). To further investigate the differences in somatic mutation frequencies among the three subtypes, we analyzed the total number of mutations after excluding samples with outlying values by means of the ‘outlier’ package in R. Our findings indicated that C1 was associated with a significantly higher mutation frequency than C2 and C3, notwithstanding a few C1 samples (**Figure 7A**). In addition, we analyzed correlations between TMB and the abundance of activated CD8^+^T cells, and between TMB and the expression of *PD-L1* and *PD1* mRNA. The abundance of activated CD8^+^ T cells and the mRNA expression of *PD-L1* and *PD1* exhibited a consistently weak positive correlation with TMB values (Pearson’s correlation coefficient test, for activated CD8^+^ T cells, R = 0.337; for PD-L1, R = 0.235; for PD-1 R = 0.135, **Figures 7B–D**). *IDH1*, *TP53*, and *ATRX* were the three most frequently mutated genes; they are involved in glycolysis, the P53 pathway, and the G2/M checkpoint, respectively (**Supplementary Figure 1**). Hallmark genes of the three pathways were identified by mining of the Molecular Signatures Database (MSigDB). The mutation frequency of *ATRX* (16.2%) was significantly lower in the C1 subtype than that in the C2 (40.7%) and C3 subtypes (24.8%). In contrast, the mutation frequency of *TP53* was significantly lower in the C3 subtype (34.8%) compared with the C1 subtype (40.5%) and C2 subtype (53.0%). Notably, the genes involved in glycolysis exhibited a higher mutation frequency relative those involved in other pathways (**Figure 7E**). Nevertheless, the three subtypes exhibited distinct mutational characteristics with respect to the three pathways.

### Diffuse Glioma Subtypes are Associated With Overall-Survival Prognosis in Different Grades of Glioma

**Figure 8:**
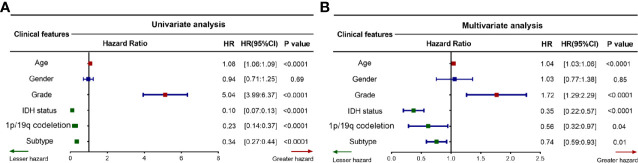
Prognostic value of the proposed subtyping for diffuse glioma. **(A, B)** Forest plots depicting the univariate Cox regression analysis **(A)** and multivariate Cox regression analysis **(B)**. The red and green curves respectively indicate poor and favorable prognostic factors, while the blue curve depicts clinical characteristics that are not associated with the prognosis. HR, hazard ratio; CI, confidence interval. *P* <0.05 was considered significant.

The above findings reveal that the three immune-related subtypes are associated with clinical outcomes. To confirm that the subtype can serve as an independent prognostic factor, we assessed the survival model in TCGA cohort using candidate independent variables ( age, gender, grade, *IDH* status, and 1p/19q codeletion). Univariate Cox regression analysis revealed that the subtype was significantly related to an improved prognosis of patients with diffuse glioma (95% confidence interval 0.27–0.44, *P* < 0.0001, **Figure 8A**). In addition, multivariate Cox regression analysis revealed that the subtype was a significant independent prognostic factor in patients with diffuse glioma, independently of age, gender, tumor grade, *IDH* mutation, and 1p/19q codeletion (95% confidence interval 0.59–0.93, *P* < 0.01, **Figure 8B**). These results collectively implied that the subtype is an independent prognostic indicator in patients with diffuse glioma. Both tumor grade and *IDH* mutation status are important independent prognostic factors of diffuse glioma. Kaplan–Meier survival curve was plotted to evaluate prognostic differences among the subtypes in LGG, higher-grade glioma (HGG), *IDH* wild-type glioma, and *IDH*-mutant glioma (**Supplementary Figure 3**). Significant prognostic differences among the three subtypes were observed in LGG and *IDH* wild-type glioma, while no significant difference was found in HGG and *IDH*-mutant glioma groups.

## Discussion

In recent years, although some approaches regarding the immune-related classification of gliomas that rely on the gene expression profiles have been proposed, no consensus has been reached on the molecular classification of diffuse gliomas, including GBM. In this study, we used immune-gene profiles to identify three distinct subtypes of diffuse glioma. To identify diffuse glioma subtypes associated with immune infiltration and clinical features, we designed a diffuse glioma classification approach based on 2,498 immune-related genes retrieved from the ImmPort database. Thus, three subtypes of diffuse glioma (C1, C2, and C3) were identified (**Figure 1**). Furthermore, we assessed subtype-specific pathways and genes, immune infiltration, clinical characteristics, and prognostic values. Our results showed that subtype-specific genes in C1 and C3 subtypes are enriched in terms associated with neutrophil-related pathways (**Figures 2A, B**). Patients with subtype C1 had a remarkably higher histological grade, a greater frequency of wild-type *IDH1* in the tumor, and higher stromal scores and immune scores than patients with subtypes C2 and C3 (**Figure 3**), and exhibited poor clinical outcomes. Moreover, five immune-cell populations (T cells, NK cells, macrophages, dendritic cells, and neutrophils) were significantly more abundant in the tumors of subtype C1 than those in the other subtypes. mRNA expression of most checkpoint proteins was found to be upregulated in subtype C1 compared with that in the other subtypes (**Figure 6**). Subtype C1 had a significantly lower mutation frequency of genes involved in glycolysis, the P53 pathway, and G2/M checkpoint (**Figure 7E**). Although the biological processes enriched in the set of subtype-specific genes of C3 are similar to those of C1, subtype C3 was characterized by a lower histological grade, lower frequency of wild-type *IDH* in the tumor, lower expression of immune checkpoint genes, lower abundance of five immune-cell populations, and lower stromal and immune scores than the other subtypes. Subtype C2 manifested clinical features intermediate between the other two subtypes. In addition, we found that the subtype is an independent prognostic indicator in patients with diffuse glioma (**Figure 8**). Overall, we noticed the heterogeneity of the immune microenvironment of diffuse gliomas of the same grade. This result partially explains why some patients with high-grade glioma have a poorer prognosis than those with low-grade glioma. Besides molecular differences, tumor-infiltrating immune cells play a critical part in the progression and treatment of glioma.

Immune cells are important components of the tumor microenvironment and perform a dual function of immunostimulation or immunosuppression, via which they either promote or inhibit the progression of tumors (34, 35). A better classification approach based on immune-related gene profiles is urgently needed for improving the efficacy of personalized immunotherapy. In this study, we found that subtype C1 contain higher abundance of immune cells, including macrophages and neutrophils. Proinflammatory cytokines and chemokines are secreted by tumor-associated macrophages (19). Tumor-associated macrophages and neutrophils are associated with a dismal prognosis in patients with diffuse glioma (36). Relevant studies have shown that patients with GBM with a high neutrophil-to-lymphocyte ratio exhibit poorer survival than those with the low ratio, probably because tumor-infiltrating neutrophils—by releasing such factors as neutrophil extracellular traps—drive the crosstalk between glioma progression and the tumor microenvironment (37). The results of these studies are consistent with our findings and partly explain why patients with subtype C1 with abundant neutrophils in the tumor have a poorer prognosis than patients with subtypes C2 and C3. In summary, we established that subtype C1 is typified by high levels of infiltration by macrophages and neutrophils, which may contribute to a poor prognosis.

Immune checkpoint receptors, including PD-1 and CTLA4, are expressed on the surface of immune cells, whereas the cognate ligands (PD-L1/PD-L2 and CD86/CD80) are expressed on the tumor cell surface. The expression of immune checkpoint receptors is commonly upregulated in the tumor microenvironment. Herein, it was demonstrated that immune checkpoint genes are differentially expressed in the three identified subtypes, in line with previous findings (38, 39). Additionally, subtype C1, with a higher proportion of GBM cases, showed higher expression of PD-L1 and PD-1 in our study. This result suggests that PD-1 checkpoint blockade can be used to identify subtype C1. Moreover, we found that PD-1 and CTLA4 are upregulated in more patients with subtype C1 GBM and are downregulated in subtype C3 GBM; these results are in agreement with the findings of another report (40). In the glioma subtypes identified here, the distribution of immune checkpoint genes was comparable to that of immune cells, including CD4^+^ T cells, activated CD8^+^ T cells, B cells, macrophages, dendritic cells, and neutrophils. Subtype C3 preferentially exhibited lower expression of immune checkpoint receptors and ligands such as PD-L1, PD-L2, CD86, and CD80. Collectively, these findings suggest that subtype C1 diffuse gliomas are characterized by a more immunosuppressive microenvironment. This hypothesis is supported by our survival analysis results, which indicate that the prognosis of patients with subtype C1 is worse than that of patients with subtypes C2 and C3. An immunosuppressive tumor microenvironment is one of the main reasons underlying chemotherapy resistance and immunotherapy failure in patients with diffuse glioma. Recent studies have shown that patients with glioma have higher expression of some immunosuppression related genes, such as *LGALS1* and *IGFBP2*, and blocking the expression of immune-inhibiting related genes can remodel the immunosuppression microenvironment (41, 42). Our study showed that patients with subtype C1 have high expression of immune checkpoint molecules in the tumor; hence, reshaping the immunosuppressive microenvironment by the blocking of immune checkpoint molecules may provide new insights into the immunotherapy for diffuse glioma and can offer a new molecular classification strategy for the accurate treatment of patients with diffuse glioma. Therefore, we can speculate that patients with subtype C1 may clinically benefit more from the blocking of checkpoint pathways. Judging by our results, certain immune cells (including CD8^+^ T cells and NK cells) that suppress tumor growth, exhibit high infiltration of subtype C1, which showed poor survival. These conflicting results suggest that the antitumor function of immune cells varies in diffuse glioma and is far more complex than previously expected. The presented immune subtyping was partially consistent with the histological grade of glioma, thereby indicating its effectiveness in terms of some aspects and its potential to supplement conventional glioma classification.

GBM patients with DNA damage repair defects may exhibit high mutation rates in the tumor that make the disease sensitive to immune checkpoint inhibitors (43). The current study showed that alterations of DNA damage repair may result in remodeling of the glioma microenvironment by modulating M2 polarization of the microglia (44). The three subtypes were found to have distinct mutation characteristics after the exclusion of outlying samples using the outlier package in R. Subtype C1 glioma has a significantly higher histological grade and mutation frequency than subtypes C2 and C3. Hypermutation in glioma is associated with CD8^+^ T cell enrichment (45). Therefore, we can theorize that more subtype C1 diffuse glioma patients are sensitive to treatment with immune checkpoint inhibitors than subtype C2 and C3 glioma patients. Some studies have revealed that major LGGs with no *IDH* mutations are molecularly and clinically similar to GBM (46). In our study, C1 had a significantly lower *IDH1* mutation frequency, thereby partly explaining why patients with subtype C1 had a dismal prognosis. Subtype C2 is associated with a markedly higher *IDH1*, *TP53*, and *ATRX* mutation frequency than the other subtypes. The prognosis of patients with subtype C2 proved to be worse than that of patients with subtype C3. This contradicts the above conclusions, which indicates that immune subtypes reflect the prognostic mechanisms of glioma better than mutations.

## Conclusions

We identified three immune-associated subtypes of diffuse glioma based on expression profiling of immune-related genes, and demonstrated the potential of the subtypes for immune checkpoint blockade. These three subtypes exhibit significant differences with respect to immune infiltration, immune checkpoint gene expression, and clinical characteristics. These findings provide novel insights into the design of immunotherapeutic strategies against diffuse glioma.

## Data Availability Statement

The original contributions presented in the study are included in the article/**Supplementary Material**, further inquiries can be directed to the corresponding authors.

## Author Contributions

QZ, CR, and XJ conceived and designed the study and analyzed the data. The other authors performed the data analysis. QZ wrote the manuscript, and XY and WL revised the manuscript. All authors contributed to the article and approved the submitted version.

## Funding

This study was supported by the National Natural Science Foundation of China [grant No. 81472355 (to XJ); grant No. 81773179 and 81272972 (to CR)] and the Hunan Provincial Science and Technology Department [grant No. 2014FJ6006 (to XJ); grant No. 2016JC2049 and 2020JJ4771 (to CR)].

## Conflict of Interest

The authors declare that the research was conducted in the absence of any commercial or financial relationships that could be construed as a potential conflict of interest.
