# Supplementary material for: Three Immune-Associated Subtypes of Diffuse Glioma Differ in Immune Infiltration, Immune Checkpoint Molecules, and Prognosis
Source: Front Oncol. 2020 Dec 23;10:586019. doi: 10.3389/fonc.2020.586019 (PMC7786360; doi:10.3389/fonc.2020.586019)
Supplement: Supplementary file 1 [file DataSheet_1.docx]

Supplementary Material

## Supplementary Figures

**
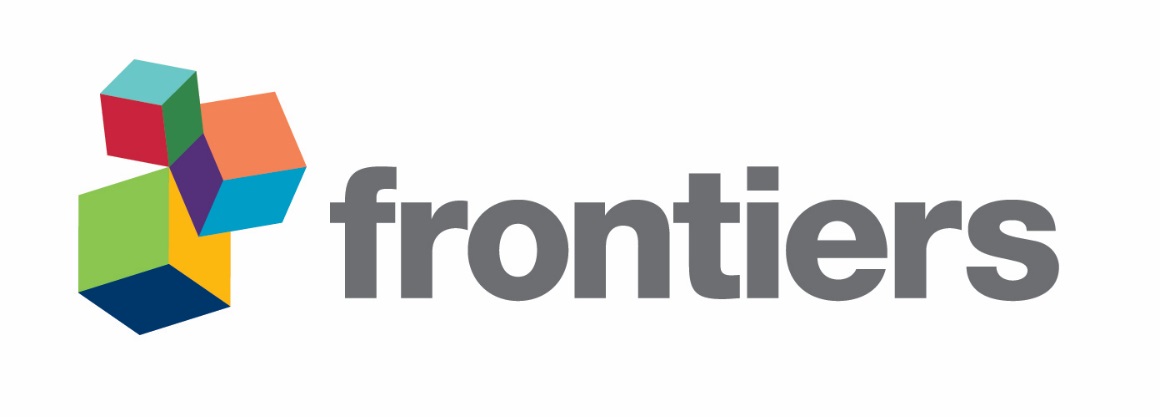
**

Supplementary Figure 1: The waterfall plot showing mutations in 25 genes with the mutation type indicated by color in the grid. Mutation rates for each sample and gene are presented in the top and left series, respectively.

Supplementary Figure 2: The expression pattern of M1 and M2 macrophages in TCGA cohorts. (A and B) Comparison of M1(A) and M2 macrophage (B) proportions by the CIBERSORT method for the three subtypes of diffuse glioma. Unpaired Student’s *t* test was performed to compare two groups with normally distributed variables, and the P values are labeled above each boxplot with asterisks (“ns” represents “not significant,” **P* < 0.05, ***P* < 0.01, ****P* < 0.001).

Supplementary Figure 3: Kaplan–Meier survival analysis of diffuse gliomas by subtype, tumor grade, and mutation status of *IDH* in the TCGA cohort. (A) *IDH*-wild-type glioma; (B) *IDH*-mutated glioma; (C) Lower-grade glioma (LGG); (D) Higher-grade glioma (HGG). The logrank test was performed to determine the significance of the differences, and data with *P* < 0.05 were considered significant.
