# Supplementary figures and images for: Three Immune-Associated Subtypes of Diffuse Glioma Differ in Immune Infiltration, Immune Checkpoint Molecules, and Prognosis
Source: Front Oncol. 2020 Dec 23;10:586019. doi: 10.3389/fonc.2020.586019 (PMC7786360; doi:10.3389/fonc.2020.586019)

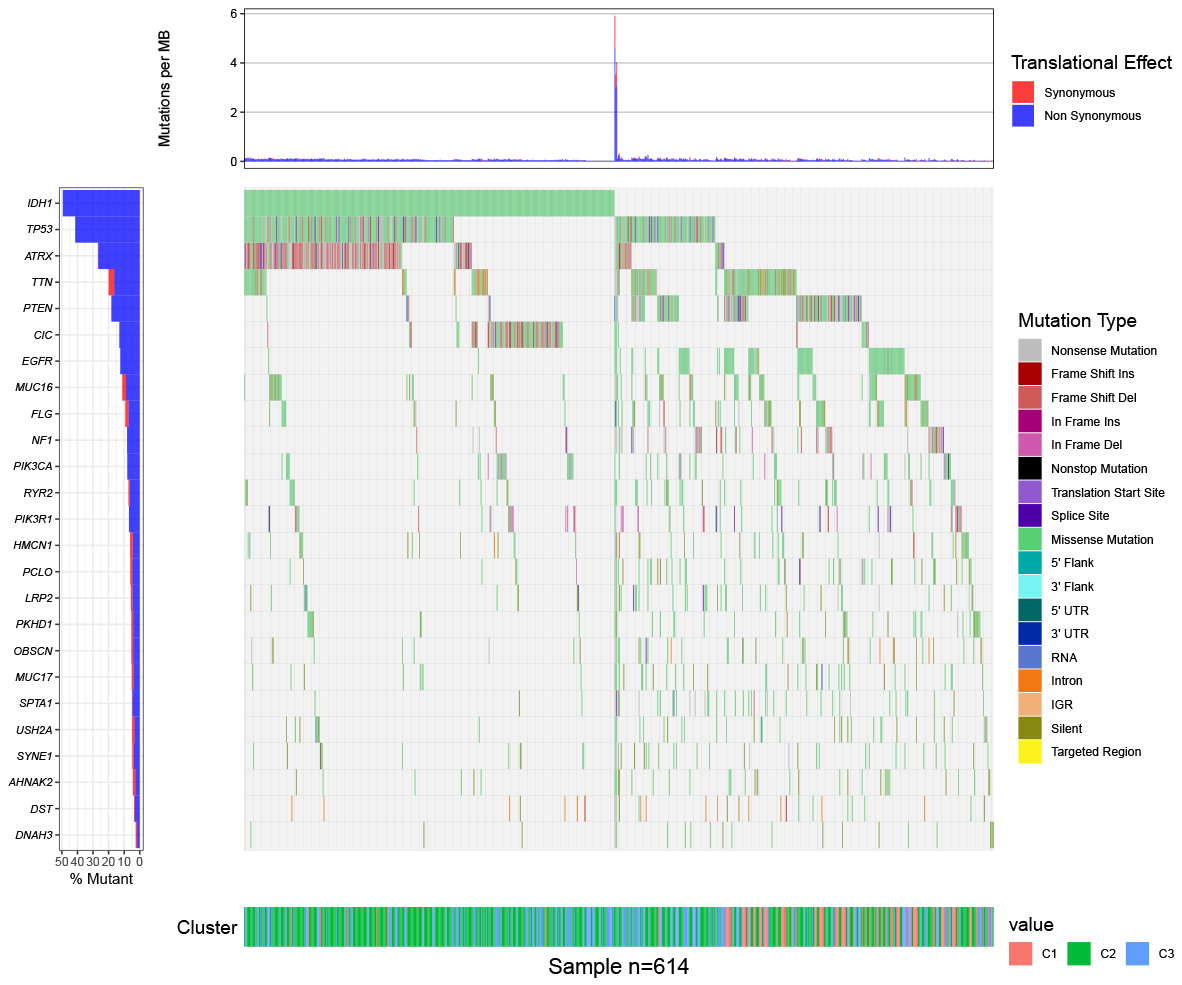

Supplement: Supplementary file 2 [file Image_1.tif]

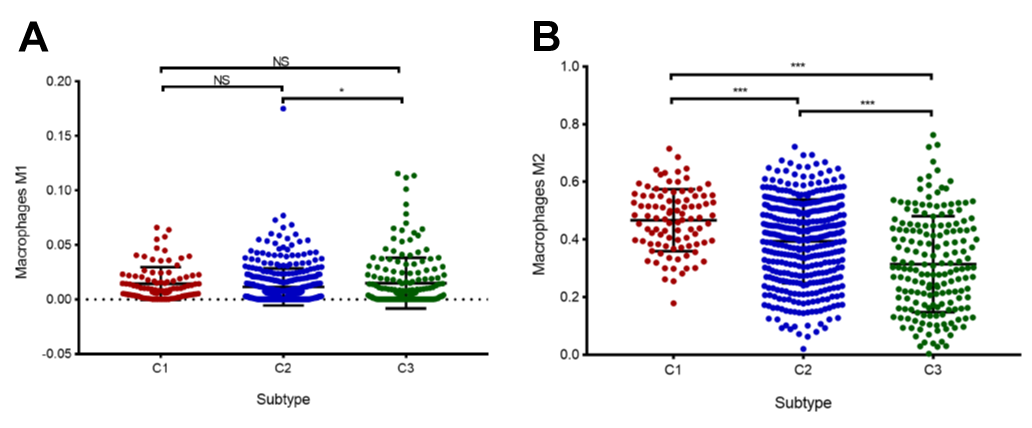

Supplement: Supplementary file 3 [file Image_2.tif]

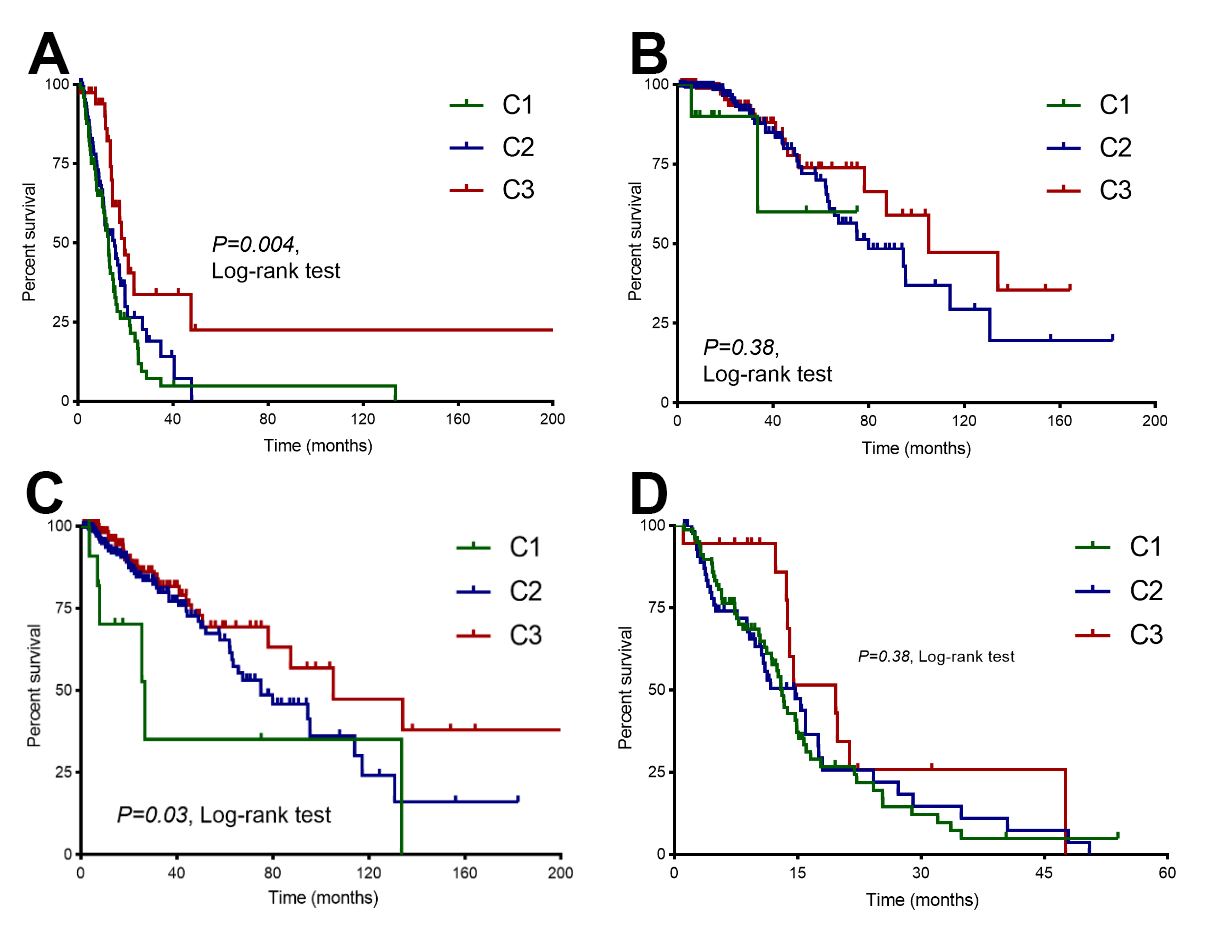

Supplement: Supplementary file 4 [file Image_3.tif]
